# A Study for Texture Feature Extraction of High-Resolution Satellite Images Based on a Direction Measure and Gray Level Co-Occurrence Matrix Fusion Algorithm

**DOI:** 10.3390/s17071474

**Published:** 2017-06-22

**Authors:** Xin Zhang, Jintian Cui, Weisheng Wang, Chao Lin

**Affiliations:** 1 Institute of Remote Sensing and Digital Earth, Chinese Academy of Sciences, Beijing 100101, China; cuijintian95@gmail.com; 2 University of Chinese Academy of Sciences, Beijing 100049, China; 3 Xinjiang Institute of Ecology and Geography, Chinese Academy of Sciences, 818 South Beijing Road, Urumqi 830011, China; wangws@ms.xjb.ac.cn; 4 Bureau of Upriver of Zhang Management, Hai River Management Committee Ministry of Water Resources of China, Handan 056006, China; almeyda05@126.com

**Keywords:** gray level co-occurrence matrix, direction measure, texture feature extraction, image classification

## Abstract

To address the problem of image texture feature extraction, a direction measure statistic that is based on the directionality of image texture is constructed, and a new method of texture feature extraction, which is based on the direction measure and a gray level co-occurrence matrix (GLCM) fusion algorithm, is proposed in this paper. This method applies the GLCM to extract the texture feature value of an image and integrates the weight factor that is introduced by the direction measure to obtain the final texture feature of an image. A set of classification experiments for the high-resolution remote sensing images were performed by using support vector machine (SVM) classifier with the direction measure and gray level co-occurrence matrix fusion algorithm. Both qualitative and quantitative approaches were applied to assess the classification results. The experimental results demonstrated that texture feature extraction based on the fusion algorithm achieved a better image recognition, and the accuracy of classification based on this method has been significantly improved.

## 1. Introduction

Texture is a description of the homogeneity of images using the texel as the fundamental unit, which has a certain scale, regularity and directionality. Texture analysis based on the local spatial variation of intensity or color brightness serves an important role in many applications of remote sensing images [1,2]. Texture analysis is extensively employed in image segmentation, classification, and pattern recognition. Moreover, texture feature extraction is an important content of texture analysis, which is an effective method for solving the problems of spectral heterogeneity and complex spatial distribution in the same category [3]. It is very critical to measure the texture reasonably and effectively, because the extracted texture features directly affect the quality of subsequent processing. At present, the methods of texture information extraction are as follows: statistical method, wavelet transform, fractal method, Markov random field (MRF) and so on. Statistical method is simple, easy to implement, and has strong adaptability and robustness. Among statistical methods, the gray level co-occurrence matrix (GLCM) is extensively applied in texture description [4], and the results from the co-occurrence matrices are better than those of other texture discrimination methods [5,6]. However, these methods have high computational complexity and lack of global information, so it is difficult to study the relationship between pixels in the texture scale. Wavelet transform and fractal method are newly developed, which extract texture information of remote sensing images based on multi-scale. The wavelet transform is suitable for various types of remote sensing data, and has a good effect on the classification of texture images with regularity and strong directionality. However, for the complex natural images, it is often ineffective due to noise interference. Fractal method processes inherently multiple-scale property and scale invariants that can be competent for roughness features in the textural representation of a texture image. Using the MRF model to describe the texture features of remote sensing images can take into account the local randomness and the overall regularity, and notes the multi-resolution of the texture, which reflects the law of geology to a great extent. However, it is mainly through model coefficients to identify the texture features; thus solving the model coefficients is difficult, and the model parameter adjustment is not convenient.

The GLCM is a classic method of texture feature extraction, which is effective in image recognition [7], image segmentation [8], image retrieval [9], image classification [10], and texture analysis methods [11,12]. The application of GLCM to extract the texture feature occurs via the joint condition probability distribution of the image gray level to represent texture and calculates the local correlation of pixels to obtain the texture feature value. The GLCM is extensively employed in many fields and has been continuously improved [13]. By calculating different directions and window sizes of the GLCM, Pacifici et al. [14] extracted multi-scale texture features from very high-resolution panchromatic imagery. Mukherjee et al. [15] preprocessed and combined the texture features by extracting from the GLCM and employed a BP-MLP (backpropagation-multilayer perceptrons) neural network to classify two types of medicinal plants. Li et al. [16] performed a principal component analysis of the image and employed the GLCM to extract texture features from the first two principal components. The texture features as a new band combined with the original image band in order to form a new image, which was employed for supervised classification. Using the texture features extracted from the GLCM, Huang et al. [17] proposed the dynamic windows algorithm to classify remote sensing imagery according to the combination of gray scale and texture features. Rao et al. [18] extracted two-order statistical parameters from the GLCM of the liquid crystal texture, including contrast, energy, uniformity and correlation, and identified the phase transition temperature of the crystal. Teng et al. [19] selected five typical texture class samples from Quick Bird data and used GLCM to quantitatively calculate six statistical texture features that were obtained by computing the average values in four directions and one pixel of pair-wise distance. The paper discussed which parameters were suitable for the specific texture classification. The above research applies GLCM to all aspects, and the texture features of images are extracted for subsequent processing, but less research takes into account the spatial directionality of texture distribution. 

Periodicity, directionality and randomness are the three most important factors in characterizing textures [20]. Therefore, the directionality of texture is a basic feature of a texture image and serves an important role for image description and understanding, which can be employed to describe the image texture [21]. Zhao et al. [22] proposed an adaptive image interpolation algorithm based on texture direction in order to improve the interpolation quality of the complex image. The four direction matrices of the image were extracted by using the Curvelet transform, which were used to obtain weight coefficients and reconstruct the value of each interpolated point. Zlatopolsky et al. [23] proposed several texture direction descriptors implemented in program LESSA (Lineament Extraction and Stripe Statistical Analysis). This described methodology was applied in different types of remote sensing image data, which obtained stable and repeatable descriptions of morphological texture. Karu [24] defined a texture that has a spatial uniform distribution of local gray-value variation and proposed a fast algorithm for mining the problem of deciding whether an image has texture. In most practical applications, the image texture features are extracted from a single direction or the mean values of the texture values in multiple directions by using GLCM. This traditional method is easy to implement, but ignores the actual directional feature of objects, especially for the effect of directionality on the texture extraction for images with distinct texture distribution. For some images with distinct texture distribution, the important information may be discarded or lost, which causes the suppression of texture directionality and disregards the spatial correlation between pattern and texture information. Therefore, the texture feature extracted from the image is not specific enough, and the classification accuracy of the texture image is limited. 

High-resolution remote sensing images have become an important source of information for all areas of geography, which usually contain spectral information and spatial structure of the two characteristics. The image spectral information is most frequently used in the interpretation and analysis of images in previous studies. However, in remote sensing images there exists the phenomenon of “same object with different spectra” and “different objects with same spectrum”. It seriously restricts the accuracy of remote sensing image analysis by only using spectral information. With the increase of the spatial resolution, the remote sensing images have richer spatial structures and texture features, which help to increase the classification accuracy [25], especially for urban environments with heterogeneous land covers. Texture directionality is a unique characteristic that was easily ignored before. Therefore, it is essential to explore the effect of the directionality of texture and accurately extract the image texture features from high-resolution remote sensing imagery for better image classification and segmentation. In order to address this problem, this paper began with investigating the directionality of the image texture extraction, aiming at reducing the suppression of texture directionality by extracting feature values from traditional methods and determining the relationship between spatial correlation and texture information, which further considered the spatial distribution and structure feature of texture, and increased the separability between objects. A novel method of texture feature extraction, based on the direction measure and the gray level co-occurrence matrix fusion algorithm, was proposed in this paper. First, according to image texture directionality, based on the method of the third-order difference, the direction measure is extracted. The direction measure describes the directional feature of the texture by calculating image gray scale changes in each direction, on this basis, structuring the weight factor. Second, the texture feature values that are extracted from different directions are combined with the weight factor. It ensures that the proportion of the texture feature values that are extracted from each direction in the final feature vector can change according to the directionality of the image, then access to the image of the final texture features. In this algorithm, the direction measure is used for texture feature extraction, which decomposes the image and reflects the similarity and regularity of the pixel gray value in different directions; the allocation of weights adaptively integrates the independent feature values of each direction, which reduces the dimension of the feature vector to four dimensions and simplifies the complexity of the research. A set of classification experiments for the high-resolution remote sensing images were performed by different methods. The experimental results demonstrated that the introduction of the direction measure effectively described the spatial association of the texture distribution and the spatial structure of complex ground objects, and it can be used to assist in the recognition of objects. Especially for the image with distinct texture, this approach proposed in the study appeared feasible and effective in image classification, which improved accuracy and reduced the complexity. 

## 2. Materials and Methods 

### 2.1. Gray Level Co-Occurrence Matrix and Two-Order Statistical Parameters

The texture is formed by the alternation of the gray scale in the spatial position, so there is a certain spatial relationship between the two pixels separated by a certain distance in the image. It is a proper way to describe the texture by studying the spatial correlation of gray scale. The gray level co-occurrence matrix (GLCM) is a common method for representing the spatial correlation of the pixel grayscale, which mainly describes the image from the interval of adjacent pixels, direction and the extent of variation. Its essence is to count the occurrence frequency of two pixels of grayscale in a certain spatial relationship, which can represent the regional consistency and relativity in the image. GLCM changes rapidly in the fine texture as the distance changes, whereas the coarse texture changes slowly. The GLCM defines a square matrix whose size represents the probability of the gray value g_1_ distanced from a fixed spatial location relationship (size and direction) to another gray value g_2_. Assume that *f*(*i*, *j*) is a 2D gray-scale image, where *S* is the set of pixels with a certain spatial relation in the region and *P* refers to the GLCM, which can be expressed as

(1) $$P(i,j)=\frac{\#\{[(i_{1},j_{1}),(i_{2},j_{2})]\in S|f(i_{1},j_{1})=g_{1}\&f(i_{2},j_{2})=g_{2}\}}{\#S}$$

Applying GLCM to describe the texture features is based on the two-order statistical parameters as the texture measure. Haralick [4] proposed 14 types of feature statistics that were based on GLCM to describe the image texture features. For remote sensing images, four types of statistics—energy (ASM), contrast (CON), correlation (COR) and entropy (ENT)—are better for texture feature extraction, and they are irrelevant between each other [26]. In this paper, the four types of feature values are employed to extract the texture features of the image (Table 1). Angular second moment (ASM) reflects the regularity and uniformity of the image distribution. Contrast (CON) reflects the depth and smoothness of the image texture structure. Correlation (COR) reflects the similarity of the image texture in a horizontal direction or vertical direction. If a texture distribution exists along a certain direction, the correlation value of the GLCM is large. Entropy (ENT) is a measure of image information, which reflects the complexity of the texture distribution.

### 2.2. Direction Measure

**Figure 1:**
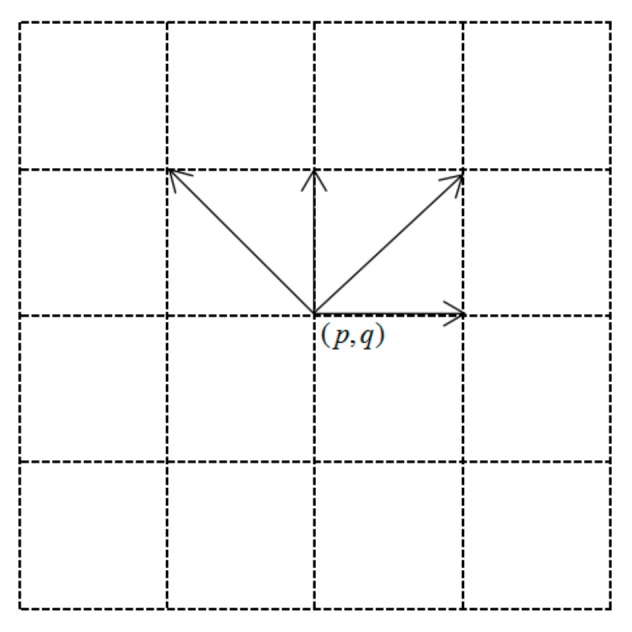
Direction measure.

The directionality of image texture is the statistic of pixel changes within a region, which reflects the similarity and regularity of the pixel gray value in different directions. According to this, the statistic of the change between the pixel gray in different directions can reflect the directional feature of texture images. The texture feature is the unity of structural characteristic in large scale and statistical characteristic in small scale. Therefore, the direction measure must reflect the structure of the image data to a certain extent, but also can reflect the statistical characteristic of the image pixels. In this paper, the direction measure statistic based on the directionality of image was constructed to describe the texture feature, which can extract the high-order statistical feature of texture, and the high-order direction measure has a high recognition rate for natural textures. The direction measure is used to decompose the image in different directions, and the change of the gray value of the image in each direction is calculated, which quantitatively yields the change rate of the image pixels in each direction. To measure the change in the neighboring pixel values in each direction, the definition of direction measure is given as follows: Assume that (*p*, *q*) is any point in the image, and the image size is *N* × *M*. Let the pixels of the image be arranged in row-major order, which lets (*p*, *q*) equal point *j* (*j* = 1, 2, 3... *N* × *M*). Then the 4 × 4 neighborhood window of the direction measure that is centered on (*p*, *q*) is shown in Figure 1, where {d(k)|k = 1, 3, 5, 7} refers to the four direction measures that are centered on (*p*, *q*), and the value of d(k) is a measure of the texture change in this direction.

The direction measures are selected as *d*(1), *d*(3), *d*(5), and *d*(7), which corresponds to 0°, 45°, 90°, 135° in Figure 1, respectively. Based on the method of the third-order difference, the equation of the direction measure is expressed as

(2) $$\left\{ \begin{array}{l} d(1)=\sum_{p=1}^{N}\sum_{q=1}^{M}\left|f(p,q+2)-3f(p,q+1)+3f(p,q)-f(p,q-1)\right|\\ d(3)=\sum_{p=1}^{N}\sum_{q=1}^{M}\left|f(p+1,q+2)-3f(p,q+1)+3f(p-1,q)-f(p-2,q-1)\right|\\ d(5)=\sum_{p=1}^{N}\sum_{q=1}^{M}\left|f(p+1,q+1)-3f(p,q+1)+3f(p-1,q+1)-f(p-2,q+1)\right|\\ d(7)=\sum_{p=1}^{N}\sum_{q=1}^{M}\left|f(p+1,q-1)-3f(p,q)+3f(p-1,q+1)-f(p-2,q+2)\right| \end{array}\right.$$

where *f*(*p*, *q*) is the gray value of the point (*p*, *q*), for the edge points of the image whose neighborhood window does not contain all pixels set to nonexistent points of the null value.

### 2.3. Fusion of Direction Measure and Gray Level Co-Occurrence Matrix

According to the calculation of direction measure, this algorithm employs the GLCM to extract the texture feature values of the image and integrates the weight factor introduced by the direction measure to ensure that the proportion of texture feature values extracted from each direction in the final feature vector can change according to the directionality of the image, then accesses to the final texture feature of the image.

#### 2.3.1. Weight Factor of Fusion Feature

Assume that (*p*, *q*) is any point in the image, and the image size is *N* × *M*; let the pixels of the image be arranged in row-major order, which lets (*p*, *q*) equal point *j* (*j* = 1, 2, 3... *N* × *M*) and *X_j_* is the gray value of point *j*. Angle *θ* = 45° × *t*, (*t* = 0, 1, 2, 3, which makes *c* = 3). $W_{tj}$ is the weight of *X_j_* that belongs to the direction measure of d(2*t* + 1)in the *θ* direction. The algorithm for determining $W_{tj}$ is expressed as

(3) $$L=\sum_{j=1}^{N\times M}\sum_{t=0}^{c}\left(w_{tj}\right)d_{tj}(x_{j},d(2t+1))$$

where *L* is the objective function.

(4) $$\left\{ \begin{array}{l} \sum_{t=0}^{c}W_{tj}=1,\forall_{j}\\ \sum_{j=1}^{N\times M}W_{tj}>0,\forall_{t}\\ W_{tj}\in [0,1],\forall_{t,j} \end{array}\right.$$

where Equation (4) is the constraint conditions.

Where $d_{tj}(x_{j},d(2t+1))=|x_{j}-d(2t+1){|}^{2}$ represents the degree of membership of *j* point, which belongs to the direction measure of *d*(*2t* + 1), and the objective function *L* is the sum of the squares of the degree of membership of *j* point, which belongs to each direction measure. Each pixel in the image belongs to a certain direction measure with different weight values, which minimizes the deviation of each pixel to its direction measure in each direction and minimizes the objective function *L*. The Lagrange multiplier method is used to obtain the optimal solution $W_{tj}$ of *L* for the constraint condition. $W_{tj}$ is expressed as

(5) $$W_{tj}=\frac{1}{\sum_{t=0}^{c}\left[d_{tj}(x_{j},d(2t+1))\right]}\times \frac{1}{\left[d_{tj}(x_{j},d(2t+1))\right]}(j=1\ldots N\times M,t=0\ldots c)$$

#### 2.3.2. Fusion Feature Calculation

$W_{t}$ is the total direction weight value of a certain direction, and the Haralick feature vectors of each direction *θ* is *Q_θ_*. The fusion feature of *Q* is calculated as

(6) $$\left\{ \begin{array}{l} W_{t}=\frac{\sum_{j=1}^{N\times M}W_{tj}}{N\times M}(\forall t,t=0,1,2,3)\\ Q_{\theta }=(ASM,CON,COK,ENT)\\ Q=\sum_{t=0}^{c}W_{t}Q_{\theta } \end{array}\right.$$

where *Q* = *(ASM*, *CON*, *COR*, *ENT)*. For this image, the fusion feature values are represented by the four fusion features.

#### 2.3.3. Steps of Fusion Feature

Four steps have been employed for feature fusions, including (1) from the upper left corner of the remote sensing image, select the kernel window with the size of *N* × *N*, and the moving step is Δ*x*, Δ*y*. For each pixel (*p*, *q*) in the image, select a certain distance, and calculate each GLCM in the direction of 0°, 45°, 90°, and 135°; (2) Based on the GLCM, the energy, contrast, correlation and entropy are calculated using the equations in Table 1. The results are taken as the texture measure of the central pixel in the window and stored in the new texture feature matrix; (3) Use Equation (2) to calculate each direction measure of the image; (4) Use Equation (6) to calculate the texture features based on the direction measure and gray level co-occurrence matrix fusion algorithm. Repeat this process until the entire image is traversed.

## 3. Experimental Results and Analysis

### 3.1. High-Resolution Remote Sensing Image Classification

The study area in this experiment is located in the Jiulong River basin, the second largest river in Fujian province. The landforms in the area are dominated by mountains, the types of land cover primarily consist of forest land and waters, and the settlements are primarily distributed along the mountains. In order to verify the effectiveness of the proposed algorithm, experiments were conducted on three types of images: the GaoFen-2 multispectral image, with 410 × 382 pixels and 4 m spatial resolution, QuickBird multispectral image, with 450 × 436 pixels and 2.44 m spatial resolution, and GeoEye-1 multispectral image, with 480 × 402 pixels and 1.65 m spatial resolution.

In this experiment, two kinds of methods were used to classify different types of images based on texture feature extraction. With the first method, the mean values of the texture values in multiple directions are employed as the image texture features. With the second method, texture feature extraction is based on the direction measure and gray level co-occurrence matrix fusion algorithm. First, we selected parameters for the GLCM in the two methods: (1) Kernel window: the kernel window of 3 × 3, 5 × 5, 7 × 7 and so on were selected and compared in order to obtain the appropriate kernel wind; (2) moving step: this experiment selected Δ*x* = 1, Δ*y* = 1 and Δ*x* = 2, Δ*y* = 2 as the moving step and made comparison. Compared with the different kernel window that obtained texture features, the smaller window can better reflect subtle changes of the image, while the larger window can better reflect the object contour and get a more vague effect. The distance between adjacent pixels is related to the image texture information, and the smaller distance between pixels can better represent the detail of the texture. Therefore, selecting the appropriate kernel window and moving step is necessary, which ensures that the image texture information can be fully extracted. By comparison, this experiment finally chose a 5 × 5 kernel window, Δ*x* = 1, Δ*y* = 1, which better reflect the coarse texture and fine texture, so we chose this size to get the calculations. Second, principal component analysis (PCA) was performed on the image in order to reduce computation and information redundancy. Through the linear transformation of the original variables, the minimal number of new variables is determined by using PCA, which can represent the data structure of the original variables to the maximum extent without losing information. The first principal component of each kind of image is used as the band which specializes in extracting the texture, and the other components are only some noise for the image information, which cannot play much effect for the image. Third, according to the characteristics of land cover in the study area, land covers were divided into six categories: forest land, arable land, water, residential land, roads, and bare land. Then, an image classification was performed based on the training samples in the study area.

A support vector machine (SVM) is a supervised machine learning algorithm that has gained popularity within visual pattern recognition and image classification [27,28]. In this experiment, the SVM classifier was used to classify the image with a combination of spectral features and texture features by using a Gaussian radial basis function (RBF). Using the same test samples, a confusion matrix was constructed for the classification results, and two types of precision evaluation indexes were established for the experimental results: the first index is to evaluate the overall accuracy (OA) of the classification by the OA and the Kappa coefficient; the second index is to measure the individual object accuracy of the classification by the conditional Kappa coefficient [29,30]. The accuracy of these two indicators was employed to compare the classification accuracy of a remote sensing image by different methods.

#### 3.1.1. GaoFen-2 Data

**Figure 2:**
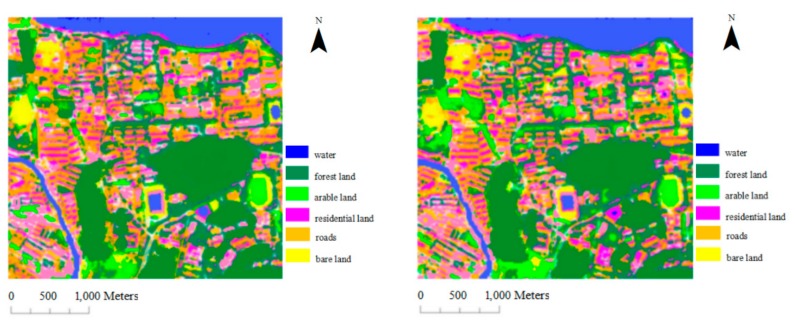
Support vector machine (SVM) classification results of GaoFen-2 data.

The multispectral image of GaoFen-2 satellites was used to classify the samples under the two methods. According to the characteristics of the image in the study area, the number of training samples (100 pixels per category) selected for each type of object was 6000, and the rest were as the test samples. The SVM classification results are shown in Figure 2, where the first image is derived by method one, and the second image is generated by method two. The overall accuracy, Kappa coefficient and conditional Kappa coefficient are shown in Table 2.

Based on the reported SVM classification accuracy of the GaoFen-2 image, the overall classification accuracy with method two has improved (92.42% vs. 86.92%), and the Kappa coefficient with method two was 0.04 greater than that with method one. The classification accuracy of arable land and water significantly increased, and the conditional Kappa coefficient increased by 0.1602 and 0.0854, respectively. Residential land and bare land increased by a lesser amount, at 0.0548 and 0.0425, respectively. No significant change was observed in the classification accuracy of forest land, which increased by only 0.0122. The classification accuracy of roads was reduced by 0.0325.

#### 3.1.2. QuickBird Data

**Figure 3:**
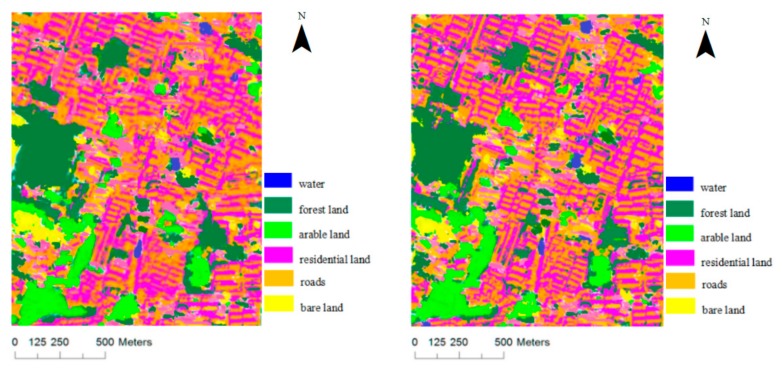
SVM classification results of QuickBird data.

The QuickBird multispectral image, with four spectral bands (red, green, blue, and near-infrared) and a 2.44 m spatial resolution, was also used to validate the proposed direction measure and gray level co-occurrence matrix fusion algorithm. In the image, the training samples (100 pixels per category) were selected for each type of object, among which forest land samples were 10,967, arable land samples were 8746, water samples were 6023, residential land samples were 6120, road samples were 5480, bare land samples were 5746, and the rest were the test samples. The SVM classification results are shown in Figure 3, where the first image is derived by method one, and the second image is generated by method two. The overall accuracy, Kappa coefficient and conditional Kappa coefficient are shown in Table 3.

Based on the reported SVM classification accuracy of the QuickBird image, the overall classification accuracy with method two has largely improved (93.26% vs. 85.70%), which was improved by 7.56%, and the Kappa coefficient with method two was 0.07 greater than that with method one. The classification accuracy of arable land and water significantly increased, and the conditional Kappa coefficient increased by 0.1316 and 0.1367, respectively. Residential land and bare land increased by a lesser amount, at 0.0674 and 0.0525, respectively. No significant change was observed in the classification accuracy of forest land and roads, which increased by only 0.0078 and 0.0045, respectively.

#### 3.1.3. GeoEye-1 Data

**Figure 4:**
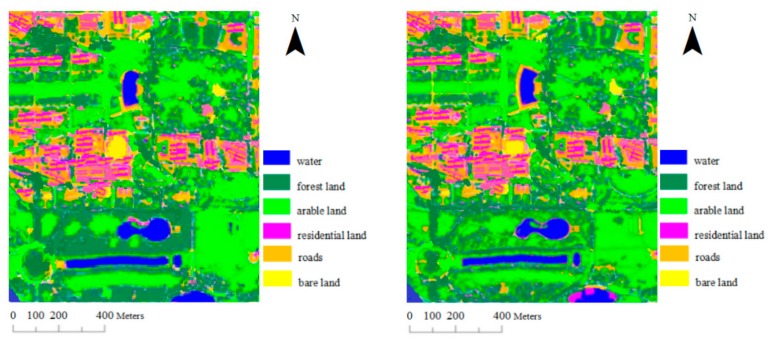
SVM classification results of GeoEye-1 data.

The proposed algorithm was further tested with the GeoEye-1 multispectral dataset from the study area. This image comprises 480 × 402 pixels, with four spectral bands (red, green, blue, and near-infrared) and a 1.65 m spatial resolution. Using the same method, we randomly chose training samples (100 pixels per category) for each type of object in the GeoEye-1 image; forest land samples were 8010, arable land samples were 9872, water samples were 5820, residential land samples were 7583, road samples were 6451, bare land samples were 5867, and the rest were as the test samples. The SVM classification results are shown in Figure 4, where the first image is derived by method one, and the second image is generated by method two. The overall accuracy, Kappa coefficient and conditional Kappa coefficient are shown in Table 4.

By analyzing the results in Table 4, it can be concluded that method two has the best effect on the classification of GeoEye-1 image with higher resolution in the classification experiments of the three types of images, the overall classification accuracy reached 96.75%, and the increase of accuracy was also the most (8.24%). According to the comparison among the conditional Kappa coefficient of various types of objects, it was similar to the first two experiments that the classification accuracy of arable land and water dramatically increased, and the conditional Kappa coefficient improved by 0.1425 and 0.1232, respectively. Residential land increased less, at 0.0645. There were basically no changes in the conditional Kappa coefficient of roads, forest land and bare land, which improved by only 0.0034, 0.0082 and 0.0095, respectively. 

By comparing the classification results of three types of high resolution remote sensing images, the following conclusions can be obtained through the above research:

Accuracies: By using the proposed direction measure and gray level co-occurrence matrix fusion algorithm, the overall classification accuracy of GaoFen-2 image (4 m spatial resolution), QuickBird image (2.44 m spatial resolution) and GeoEye-1 image (1.65 m spatial resolution) were 92.43%, 93.26% and 96.75%, respectively, which were improved by 5.51%, 7.56%, and 8.24%, respectively, by comparison of method one. Also, for the increase of Kappa coefficient, the GeoEye-1 image improved the most, followed by the QuickBird image and GaoFen-2 image. It is interesting to see that when the spatial resolution of the image increases, using the fusion algorithm to extract the texture values to participate in the classification, the overall accuracy of the image classification is higher, and it has better classification results than the mean values of the texture values. Therefore, it can be deduced that with the increase in spatial resolution of image and the refinement of classification method, the morphological distribution within the class will become more concentrated and concrete, and the differences between the classes are more significant. The introduction of direction measure increases the validity of texture feature values, thereby increasing the separability of objects. 

Comparison: For the conditional Kappa coefficient of different objects, in the experiment of the GaoFen-2 image, the classification accuracy was improved obviously in arable land and water, slightly in residential land and bare land, basically no change in forest land, and the conditional Kappa coefficient of roads was reduced; in the same experiment carried out in the QuickBird image, it can be summarized that the classification accuracy of arable land and water significantly improved, residential land and bare land increased by a lesser amount, and no significant change was noticed in the classification accuracy of forest land and roads; the further test with the GeoEye-1 image showed it was similar to the first two experiments in that the classification accuracy of arable land and water dramatically increased; however, residential land of that increased less, and the conditional Kappa coefficient of roads, forest land and bare land changed slightly. Using the algorithm proposed in this paper, the classification accuracy of arable land and water has been greatly improved in the three types of image experiments. The arable land belongs to the land use type of human transformation, which is adjacent to each other in the spatial distribution, and the shape is regular; from the characteristics of the ground features, the surface of the water body is smooth and continuous, the fluctuation is small and the texture is fine. The surface roughness of the water area is significantly lower than that of other ground objects, and the change of gray scale in the water image is relatively continuous. Due to the characteristics of water body texture, the water area is extremely homogeneous in the image. Therefore, the texture values of these two types of objects can reflect the slight changing regularity of the direction, and the classification results have been significantly improved by using the fusion algorithm proposed in this paper. Through the analysis of the above experimental results, using the fusion algorithm proposed in this paper for classification is more accurate, especially for the image texture distribution with a certain regularity and directionality. For other objects, due to the difference in spatial resolution, image spectrum, characteristics of object structure and so on, the change of the conditional Kappa coefficient varies. However, the introduction of directionality has not much of a negative impact on the classification accuracy. 

### 3.2. Direction Measure of Image

**Figure 5:**
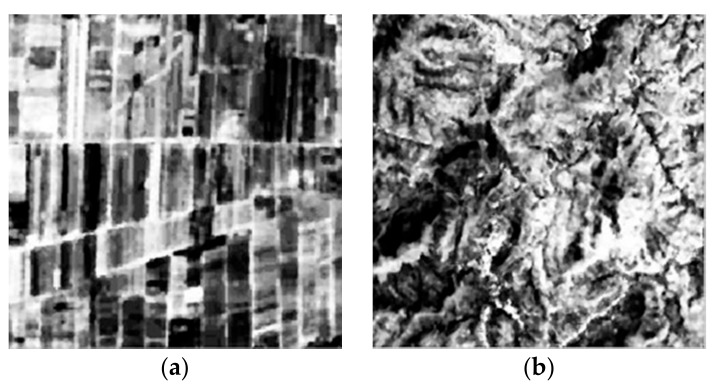
Different texture types on a remote sensing image ((**a**)-arable land; (**b**)-forest land).

Two different types of textures of the first principal component GaoFen-2 image after preconditioning were selected: arable land with a distinct texture direction (Figure 5a) and forest land without a distinct texture direction (Figure 5b). Using the algorithm developed in this paper, we calculated two types of image direction measures and their corresponding weight factors (Table 5).

As shown in Table 5, the texture direction of the high-resolution remote sensing image of the arable land selected in this experiment was 90°. Thus, the direction measure of 90° was the smallest, and the weight of the texture feature values in the direction was the largest. Compared with arable land, the texture changes of forest land in the four directions were not distinct, and the difference of each direction measure was not significant. This experiment verified that the direction measure can measure the gray scale change of the image, which can measure whether the texture image distributes in a certain direction. By comparing the relative values of the direction measure, the direction corresponding to the minimum measurement is generally the texture direction of the image, and the weight of this direction is relatively large. Conversely, the value of each direction measure is similar, and the weight of each direction tends to be evenly distributed. It indicates that when a significant difference is observed between the measured value of one direction and the measured values of other directions, the image texture has a distinct direction; otherwise, the image texture is uniform.

### 3.3. Image Classification with or without Distinct Directionality

According to the previous two experiments, the classification accuracy of arable land with a distinct texture direction was improved, and forest land without a distinct texture direction exhibited no change in the classification results of the SVM. Therefore, this experiment selected a typical sample of arable land and forest land images in the first principal component of GaoFen-2 image after pretreatment, each of which included 100 pieces. The KNN (k-nearest neighbor) classifier, a machine learning classification method, was employed for the classification of images with or without a distinct texture direction, and the accuracy of method one was compared with the accuracy of method two. The input of KNN is the feature vector of samples, and the output is the type of samples. KNN assumes that a training dataset is given, in which the sample class has been determined and classified, and the new samples are predicted by the majority of the k samples of the nearest neighbor training samples.

Methods one and two were used to extract the texture feature values of forest land and arable land, including energy (ASM), contrast (CON), correlation (COR), and entropy (ENT). The results of the two methods are shown in Table 6 and Table 7. Using MATLAB R2014a as the experimental platform, the KNN classifier was edited, and the images were divided into a training set and a test set, respectively. After the size of the selected image was normalized, the size of each sample was 150 × 150 pixels, and all samples were numbered. The first 50 sub-samples were employed as the training set, and the latter 50 sub-samples were employed as the test set. Therefore, the total number in the training set was 100, and the total number of test samples was 100. The training set was trained and the test set was classified by the KNN classifier. Using cross-validation to select the optimal k value and the KNN classifier based on Euclidean distance, the samples were input into the KNN classifier to verify the classification accuracy of the two methods (Table 8).

Different methods were used to extract the texture features as the input data of the KNN classifier, and the experimental results were different. By comparing Table 6 and Table 7, the differences among the texture feature value in the different categories extracted by method two were more distinct than the texture feature value in the different categories extracted by method one. The weighting factor obtained from the direction measure further modified the texture features value in the multiple directions obtained by the GLCM. As shown in Table 8, using method one and method two, which had minimal impact on the classification accuracy, the latter increased by 0.88% for the forest land without a distinct texture direction. For arable land with a distinct texture direction, the method proposed in this paper achieved a classification accuracy of 95.46% and an increase in accuracy by 4.21% compared to method one, which demonstrated the results of experiment 1 and experiment 2.

## 4. Discussion

The directionality of texture contains a large amount of visual information and image spatial distribution information, which is an effective approach to texture representation and graphical modeling. Due to the image texture distribution with a certain regularity and directionality, the direction measure statistic, which is based on the directionality, may be essential for image classification, especially for heterogeneous urban environments. This paper is aimed at reducing the suppression of texture directionality by extracting feature values from traditional methods and obtaining the relationship between spatial correlation and texture information. A new algorithm that is based on the fusion of direction measure and the gray level co-occurrence matrix (GLCM) was proposed in this paper, which can better describe the texture features in terms of local detail information and spatial distribution. The direction measure divides the image, and maintains the geometric invariance and optical invariance of the image while measuring the directionality of the image. Specifically, we compared the methods (i.e., fusion algorithm and the mean values of the texture values in multiple directions) to classify the high-resolution image with the combination of spectral features and texture features. In general, the classification combined with texture features by using the direction measure and gray level co-occurrence matrix fusion algorithm has achieved good results, which was better than that of using the SVM classifier based on the mean values of the texture values extracted from the GLCM in multiple directions. The overall classification accuracy and the Kappa coefficient with fusion algorithm have largely improved, and the experimental results verified the effectiveness of the direction measure. By combining with the direction measure of texture and original images, it can achieve a stronger image recognition, enhance the boundary, and highlight the color and contour of various objects in the original images, which plays a complementary role on the gray value of the images. Basically, all of these can provide a better external condition for the improvement of classification accuracy. 

In addition, the direction measure calculates the gray-scale change of the image, which can measure whether the texture image distributes in a certain direction. Using the algorithm developed in this paper, we calculated two types of image direction measures and their corresponding weight factors. This test demonstrated that when a significant difference is observed between the measured value of one direction and the measured values of other directions, the image texture has a distinct direction; otherwise, the image texture is uniform. Moreover, in KNN tests, extracting the feature values of different samples with different methods was used as the input of the KNN classifier. The results indicated that the differences among the texture feature values in the different categories extracted by fusion algorithm were more distinct, which showed a satisfactory performance in classification.

The study of image texture extraction and texture directionality has the following three advantages: (1) As a kind of important spatial structure, texture feature includes the surface information of the feature and its relationship with the surrounding environment, which better balances the image of the macro-structure and micro-structure. Therefore, the classification of remote sensing images combined with texture information can help to suppress the impact of “same object with different spectra” and “different objects with same spectrum”, and improve the classification accuracy; (2) The extraction of texture directionality reflects the objective information of various objects in the remote sensing image, which further distinguishes and describes the characteristics of different objects, and provides a better external condition for classification; (3) Considering that the majority of the image applications are related to texture, especially for some objects with distinct texture distribution, the proposed algorithm in this study is more practical. It can be widely applied to fringe analysis, force analysis, calculation of the direction and intensity of erosion, prediction of satellite cloud image and so on, which has a very practical significance for the understanding of images.

However, there are still some problems to be further studied in the experiment. Natural texture images inevitably have a small inconsistency in direction, which may have some influences on the texture extraction based on direction. Especially when the direction consistency requirement is not fully satisfied, the compactness of the data points in the feature space is reduced and the clustering effect is affected. Therefore, the next step is to study the robustness of the texture direction change and the image preprocessing method before extracting texture features to achieve satisfactory results. 

## Figures and Tables

**Table 1:** Two-order statistical parameters.

Method	Equation	Description
Angular second moment (ASM)	$ASM=\sum_{i}^{N}\sum_{j}^{N}P(i,j{)}^{2}$	
Contrast (CON)	$CON=\sum_{i}^{N}\sum_{j}^{N}{\left(i-j\right)}^{2}P(i,j)$	
Correlation (COR)	$COR=\frac{\sum_{i}^{N}\sum_{j}^{N}\left(i-\bar{x})(j-\bar{y})P(i,j\right)}{\sigma_{x}\sigma_{y}}$	$\begin{array}{c} \bar{x}=\sum_{i}^{N}i\sum_{j}^{N}P(i,j)\;\bar{y}=\sum_{j}^{N}j\sum_{i}^{N}P(i,j)\\ {\sigma_{x}}^{2}=\sum_{i}^{N}{\left(i-\bar{x}\right)}^{2}\sum_{j}^{N}P(i,j)\\ {\sigma_{y}}^{2}=\sum_{j}^{N}{\left(j-\bar{y}\right)}^{2}\sum_{i}^{N}P(i,j) \end{array}$
Entropy (ENT)	$ENT=-\sum_{i}^{N}\sum_{j}^{N}P(i,j)\mathrm{lg}P(i,j)$	

**Table 2:** SVM classification accuracy of GaoFen-2 data.

Class	Method One	Method Two
Water	0.8681	0.9535
Forest land	0.8543	0.8665
Arable land	0.8374	0.9976
Residential land	0.7016	0.7564
Roads	0.7113	0.6788
Bare land	0.5974	0.6399
OA/%	86.92	92.43
Kappa coefficient	0.83	0.87

The accuracy of each class in the table is the conditional Kappa coefficient.

**Table 3:** SVM classification accuracy of QuickBird data.

Class	Method One	Method Two
Water	0.8425	0.9792
Forest land	0.8517	0.8595
Arable land	0.8526	0.9842
Residential land	0.6937	0.7611
Roads	0.7012	0.7057
Bare land	0.6843	0.7368
OA/%	85.70	93.26
Kappa coefficient	0.82	0.89

The accuracy of each class in the table is the conditional Kappa coefficient.

**Table 4:** SVM classification accuracy of GeoEye-1 data.

Class	Method One	Method Two
Water	0.8714	0.9946
Forest land	0.8623	0.8705
Arable land	0.8435	0.9860
Residential land	0.7123	0.7768
Roads	0.6930	0.6964
Bare land	0.6551	0.6646
OA/%	88.51	96.75
Kappa coefficient	0.84	0.93

The accuracy of each class in the table is the conditional Kappa coefficient.

**Table 5:** Direction measure and weight factor of different texture images.

	Arable Land				Forest Land			
	0°	45°	90°	135°	0°	45°	90°	135°
Direction measure	133	94	12	67	64	75	61	70
Weight factor	0.017	0.032	0.864	0.087	0.257	0.208	0.311	0.224

**Table 6:** Method one for texture feature extraction.

Class	ASM	CON	COR	ENT
Arable land	0.0447	3.8586	0.0481	4.2301
	0.0411	3.0875	0.0389	4.5788
	0.0439	3.1024	0.0431	4.4029
	0.0392	2.9304	0.0379	4.5967
Forest land	0.2670	2.6334	0.1446	2.4026
	0.2040	2.2045	0.1384	2.4786
	0.2134	2.1342	0.1265	2.7665
	0.2587	2.4563	0.1323	2.5876

**Table 7:** Method two for texture feature extraction.

Class	ASM	CON	COR	ENT
Arable land	0.0556	6.2471	0.0470	5.4875
	0.0549	6.0083	0.0413	6.0032
	0.0551	6.1267	0.0434	5.8976
	0.5127	5.9872	0.0405	6.1233
Forest land	0.3456	1.2545	0.2854	2.2321
	0.3208	1.2074	0.2475	2.2482
	0.3306	0.1944	0.2409	2.4874
	0.3418	1.2475	0.2463	2.3677

**Table 8:** Accuracy of remote sensing image classification with or without distinct texture.

Method	Forest Land	Arable Land
Method one	90.57	91.30
Method two	91.45	95.46
